# Non-alcoholic fatty liver disease, vascular inflammation and insulin resistance are exacerbated by TRAIL deletion in mice

**DOI:** 10.1038/s41598-017-01721-4

**Published:** 2017-05-15

**Authors:** Siân P. Cartland, Hanis H. Harith, Scott W. Genner, Lei Dang, Victoria C. Cogger, Melissa Vellozzi, Belinda A. Di Bartolo, Shane R. Thomas, Leon A. Adams, Mary M. Kavurma

**Affiliations:** 1 Heart Research Institute, Sydney, 2042 Australia; 2 The University of Sydney, Sydney Medical School, Sydney, 2006 Australia; 3 University of New South Wales, School of Medical Sciences, Sydney, 2052 Australia; 4 Universiti Putra Malaysia, Department of Biomedical Science, Faculty of Medicine and Health Sciences, Selangor, 43400 Malaysia; 5 The University of Sydney, Charles Perkins Centre, Sydney, 2006 Australia; 6 ANZAC Research Institute and Ageing and Alzheimers Institute, Concord Hospital, Sydney, 2139 Australia; 7 The University of Western Australia, School of Medicine and Pharmacology, QEII Medical Centre Unit, Crawley, 6009 Australia

## Abstract

Non-alcoholic fatty liver disease (NAFLD) incorporates steatosis, non-alcoholic steato-hepatitis (NASH) and liver cirrhosis, associating with diabetes and cardiovascular disease (CVD). TNF-related apoptosis-inducing ligand (TRAIL) is protective of CVD. We aimed to determine whether TRAIL protects against insulin resistance, NAFLD and vascular injury. Twelve-week high fat diet (HFD)-fed *Trail* ^−*/*−^ mice had increased plasma cholesterol, insulin and glucose compared to wildtype. Insulin tolerance was impaired with TRAIL-deletion, with reduced p-Akt, GLUT4 expression and glucose uptake in skeletal muscle. Hepatic triglyceride content, inflammation and fibrosis were increased with TRAIL-deletion, with elevated expression of genes regulating lipogenesis and gluconeogenesis. Moreover, *Trail* ^−*/*−^ mice exhibited reduced aortic vasorelaxation, impaired insulin signaling, and >20-fold increased mRNA expression for IL-1β, IL-6, and TNF-α. *In vitro*, palmitate treatment of hepatocytes increased lipid accumulation, inflammation and fibrosis, with TRAIL mRNA significantly reduced. TRAIL administration inhibited palmitate-induced hepatocyte lipid uptake. Finally, patients with NASH had significantly reduced plasma TRAIL compared to control, simple steatosis or obese individuals. These findings suggest that TRAIL protects against insulin resistance, NAFLD and vascular inflammation. Increasing TRAIL levels may be an attractive therapeutic strategy, to reduce features of diabetes, as well as liver and vascular injury, so commonly observed in individuals with NAFLD.

## Introduction

Non-alcoholic fatty liver disease (NAFLD) is the most common liver condition in the Western world and an increasingly common indication for liver transplantation^1, 2^. It incorporates a spectrum of liver diseases characterized by abnormal accumulation of lipid (steatosis) arising in the absence of excess alcohol consumption. These diseases cover a continuum of stages ranging from steatosis, to nonalcoholic steatohepatitis (NASH) and cirrhosis, eventually resulting in liver failure. NAFLD is strongly associated with type-2 diabetes (T2D), with NASH patients displaying hepatic insulin resistance, inflammation and fibrosis^3^. NAFLD is not only associated with metabolic syndrome but is also an independent risk factor for cardiovascular disease (CVD), with CVD accounting for majority of deaths in patients with this disease^3^. Understanding the molecular and cellular hepatic changes that occur with progressive NAFLD will lead to the development of more sophisticated treatment options for these people.

Tumor necrosis factor (TNF)-related apoptosis inducing ligand (TRAIL) is a membrane bound and soluble cytokine found throughout the body. Originally identified because of its high sequence homology to TNF-α and Fas ligand (FasL), TRAIL can promote apoptosis and non-apoptotic pathways^4–7^, however, its role *in vivo* in normal physiology is unclear. TRAIL is implicated in the pathogenesis of CVD and diabetes with circulating levels reduced in people with disease^8, 9^. Importantly, TRAIL-deletion in *Apoe* ^−/−^ mice leads to increased plasma cholesterol and glucose, accelerating atherosclerosis and features of diet-induced diabetes^10^; risk factors for NAFLD. The extent to which TRAIL protects against NAFLD is unclear. In this study, we examined (i) levels of circulating TRAIL in patients with increasing NAFLD severity; (ii) NAFLD pathogenesis using murine models of TRAIL-deletion; and (iii) the direct effect of TRAIL in NAFLD *in vitro*. Here we show that TRAIL protects against NASH, and may be a promising therapeutic for the treatment of NAFLD and associated pathologies.

## Results

### Patients with NASH have reduced circulating TRAIL strongly associating with plasma ALT

**Figure 1:**
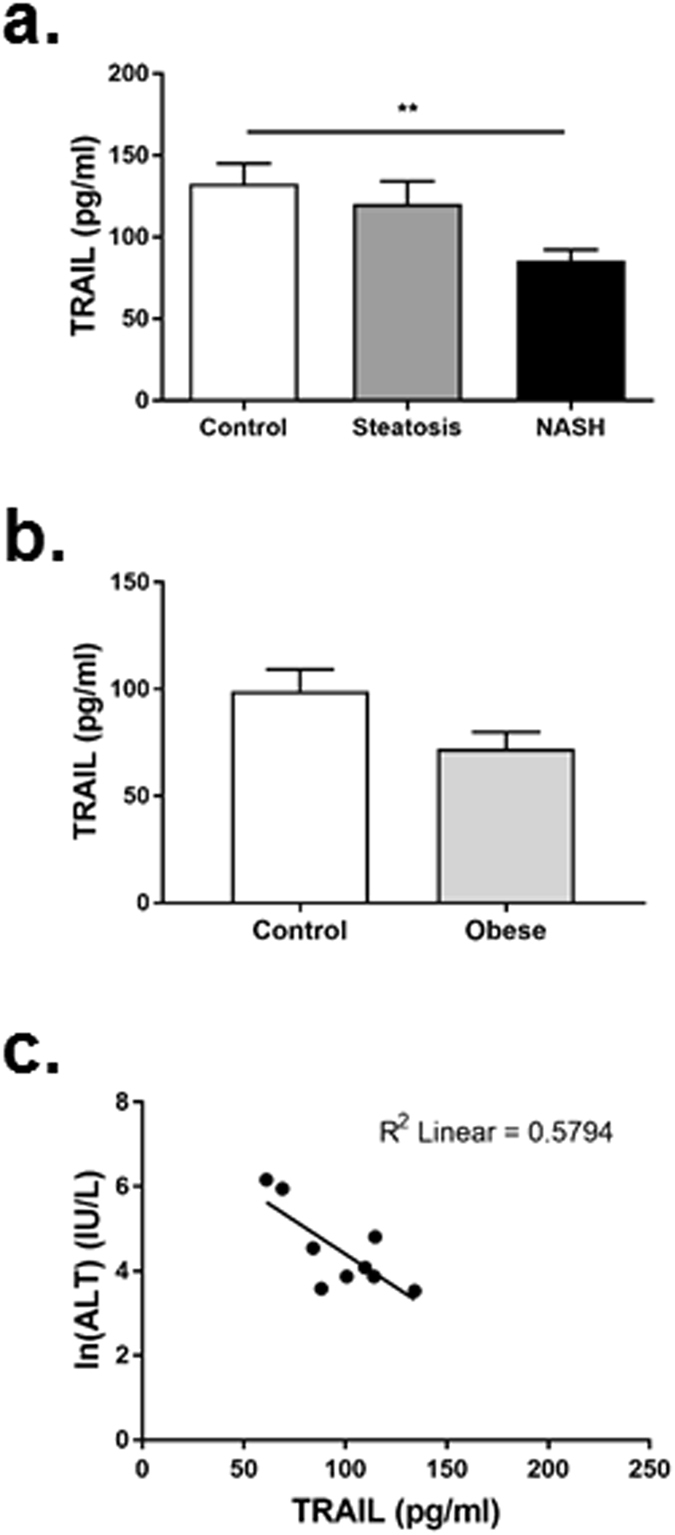
Patients with NASH have reduced circulating TRAIL. (**a**) Circulating TRAIL levels in control, steatosis or NASH patients. (**b**) Circulating TRAIL levels in control and obese individuals. (**c**) Correlation between plasma TRAIL and ALT levels (Spearman rho −0.516, p = 0.003). Natural log (ln) of serum ALT presented graphically. n = 9–10/group; ANOVA and Mann Whitney *U* test.

Clinical data are summarized in Table 1. Patients with NASH had significantly increased body mass index (BMI), waist circumference and were diabetic, compared to individuals with simple steatosis. NASH patients also had significantly elevated plasma alanine transaminase (ALT), aspartate aminotransferase (AST) and triglycerides, with reduced high-density-lipoprotein (HDL)-cholesterol. Histologically, increased steatosis, inflammation, ballooning and fibrosis were observed with NASH; and consistent with the histological diagnosis of NASH, the NAFLD activity score (NAS) was >5 in these individuals. Importantly, circulating TRAIL levels were significantly reduced in patients with NASH compared to controls, but not simple steatosis (Fig. 1a). There was no change in circulating TRAIL between control and obese individuals (Fig. 1b). Further, we examined the correlation between plasma TRAIL levels and multiple metabolic, clinical and biochemical parameters including age, BMI, serum glucose, insulin, triglyceride, HDL-cholesterol and AST levels (Supplemental Table 1), and found no associations with TRAIL and these metabolic, clinical or biochemical variables. However, a strong negative correlation between plasma TRAIL and ALT was observed in NASH patients (Fig. 1c). Serum ALT remained significantly associated with plasma TRAIL levels after adjustment for diabetes using linear regression analysis (beta −0.504, *p* = 0.004). The relationship remained robust and significant following further adjustment for BMI, age and sex (beta −0.461, *p* = 0.01). These findings indicate that with liver injury, independently of diabetes, circulating TRAIL levels are reduced and inversely associate with plasma ALT.

**Table 1:** Clinical and biochemical characteristics of control, simple steatosis and NASH patients.

	Normal (n = 9)	Obese (n = 10)	Simple steatosis (n = 10)	NASH (n = 10)	*P* value
Age (years)	48.9 (8.7)	40.0 (9.5)	39.1 (12.1)	52.0 (14.9)	0.11
Male (n, %)	4 (44%)	0 (0%)	4 (40%)	4 (40%)	0.009
BMI (kg/m^2^)	24.6 (2.7)	38.9 (6.0)	36.2 (8.6)	34.1 (4.6)	0.001
Waist Circumference (cm)	82 (8)	107 (19)	99 (39)	118 (15)	0.02
Diabetes (n, %)	0 (0%)	0 (0%)	1 (10%)	7 (70%)	0.001
ALT (IU/l)	26 (8)	32 (8)	55 (29)	159 (163)*	0.003
AST (IU/l)	27 (7)	18 (4)	23 (14)	86 (95)*	0.01
Glucose (mmol/l)	5.0 (0.5)	4.9 (0.4)	4.4 (3.3)	7.9 (5.2)	0.07
Insulin (U/l)	3.8 (1.3)	14.1 (9.3)	12.1 (14.1)	31.4 (49.7)*	0.15
Triglyceride (mmol/l)	0.9 (0.3)	1.0 (0.3)	1.5 (1.2)	3.0 (1.5)*	0.001
HDL-Cholesterol (mmol/l)	1.6 (0.2)	1.4 (0.4)	1.3 (0.4)	0.9 (0.1)*	<0.001
Total Cholesterol (mmol/l)	4.9 (0.8)	5.4 (1.1)	4.7 (1.7)	4.5 (1.3)	0.56
Steatosis	—	0	1.7 (0.7)	2.5 (0.5)	0.01
Inflammation	—	0	0.0 (0.0)	1.3 (0.5)	<0.001
Ballooning	—	0	0.0 (0.0)	1.6 (0.5)	<0.001
NAS	—	0	1.7 (0.7)	5.4 (0.8)	<0.001
Fibrosis	—	0	0.0 (0.0)	2.1 (0.9)	<0.001

Footnote: Continuous data presented as mean (standard deviation). Comparison between groups performed using Chi-squared test, or ANOVA with comparison between Simple Steatosis and NASH groups with Bonferroni correction, apart from histological parameters, which are compared using Mann-Whitney *U* test. *p < 0.05 for post-hoc comparison between simple steatosis and NASH patients.

### NAFLD reduces circulating TRAIL and alters TRAIL receptor expression *in vitro* and *in vivo*

**Figure 2:**
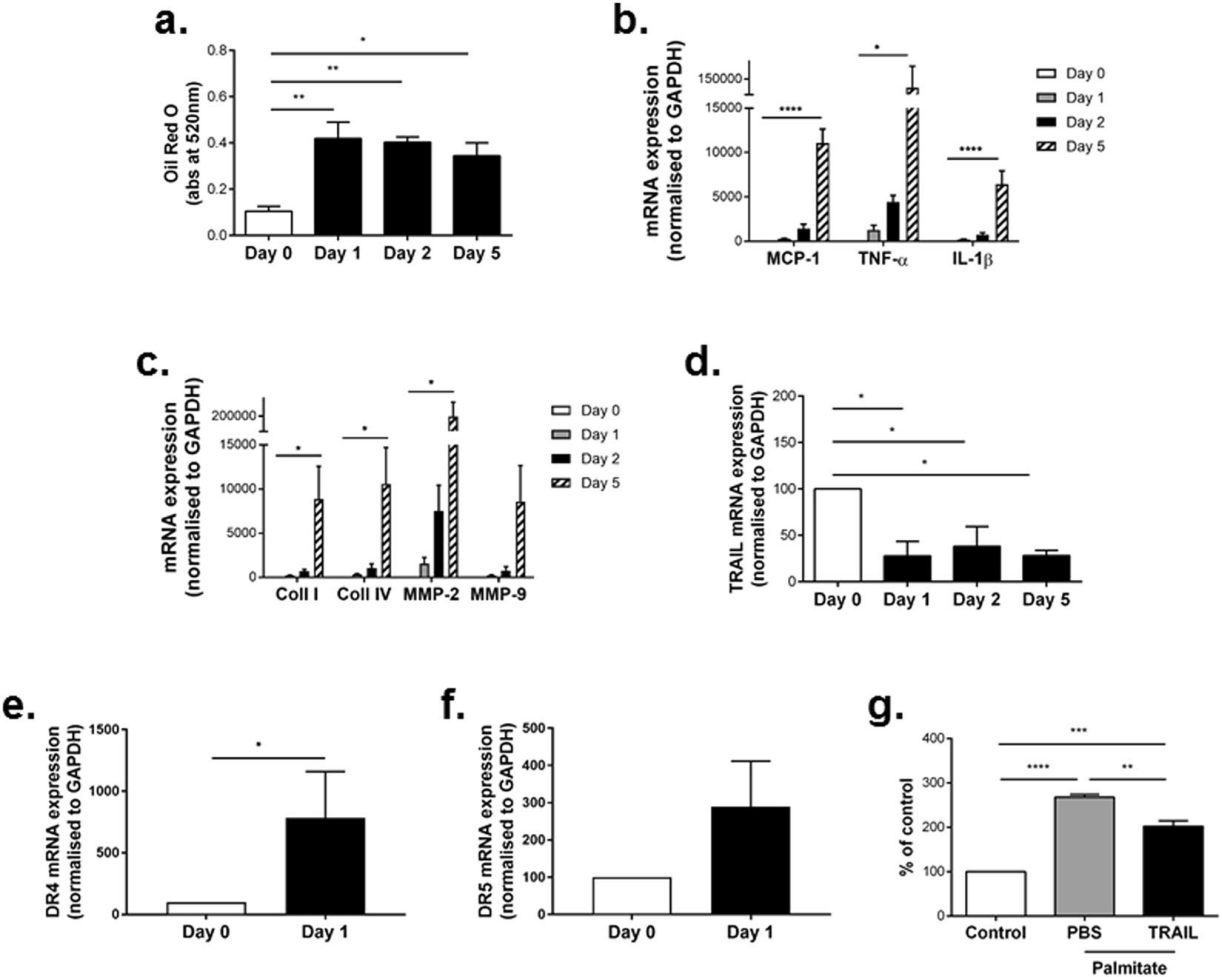
TRAIL protects against lipid uptake in HepG2 cells. (**a**) Palmitate increases triglyceride uptake in HepG2 cells over 5 days as measured by Oil red O. (**b**) Inflammation and (**c**) fibrotic markers are increased in HepG2 cells in response to palmitate over 5 d. (**d**) mRNA expression for TRAIL is significantly reduced with lipid uptake over time. (**e**) DR4 and (**f)** DR5 expression in response to palmitate. (**g**) 1 ng/ml TRAIL inhibits palmitate-induced oil red O in HepG2 cells at 24 h compared to the vehicle PBS. Control, untreated cells; n = 3–4/treatment. mRNA expression was normalized to GAPDH. Results are expressed as mean ± SEM; ANOVA; *p < 0.05, **p < 0.01, ***p < 0.001 and ****p < 0.0001.

To directly delineate the effect of free fatty acids on hepatocytes and TRAIL expression, we used an *in vitro* model of NAFLD^11^. As expected, treatment of HepG2 cells with BSA-conjugated palmitate significantly increased oil red O staining, which is indicative of triglyceride content (Fig. 2a). This increase in triglyceride-content was also associated with increased expression of inflammatory and fibrotic markers (Fig. 2b,c). In contrast, lipid loading significantly reduced (~50%) TRAIL mRNA expression (Fig. 2d), while DR4 and DR5 expression was elevated, with only DR4 reaching significance (Fig. 2e,f). Importantly, exogenous TRAIL treatment of hepatocytes significantly reduced palmitate-induced lipid uptake *in vitro* by ~30% (Fig. 2g).

Wildtype mice fed a HFD for 12 w develop NAFLD^12^. In response to a HFD, wildtype mice exhibited significant increases in body weight, fasting plasma glucose, insulin and total cholesterol (Supplemental Table 2). Importantly, circulating TRAIL levels were reduced by almost 3-fold (Supplemental Fig. 1a). In contrast to hepatocytes *in vitro*, a significant increase in hepatic TRAIL expression was observed in 0 vs. 12 w HFD wildtype mice (Supplemental Fig. 1b), which associated with a significant reduction in hepatic mDR5 mRNA (Supplemental Fig. 1c). Similarly, TRAIL levels were increased in white adipose tissue (WAT), with mDR5 expression also increased (Supplemental Fig. 1d,e). These data demonstrate that changes to TRAIL signals in hepatocytes *in vitro*, do not reflect the changes observed in the complex setting of a fatty liver of wildtype mice *in vivo*.

### *Trail*^−*/*−^ mice display altered plasma chemistries

To assess whether TRAIL deletion alone affects metabolic changes, 12 w HFD wildtype were compared with 12 w HFD *Trail* ^−*/*−^ mice. In contrast to our previous findings in *Trail* ^−*/*−^ *Apoe* ^−/−^ vs. *Apoe* ^−/−^ mice^10^, there was no observed difference in body weight (Supplemental Table 2), food intake or total energy expenditure (Supplemental Table 3) between wildtype and *Trail* ^−*/*−^ mice. 12 w HFD *Trail* ^−*/*−^ mice did however, display an increase in rearing activity compared to wildtype (Supplemental Table 3). Importantly, *Trail* ^−*/*−^ had elevated plasma glucose, insulin and cholesterol levels, with a significant reduction in plasma triglycerides when compared to 12 w HFD wildtype mice (Supplemental Table 3). Collectively these findings indicate that HFD-fed *Trail* ^−*/*−^ mice have a more profound T2D phenotype.

### *Trail*^−*/*−^ mice have impaired insulin sensitivity

**Figure 3:**
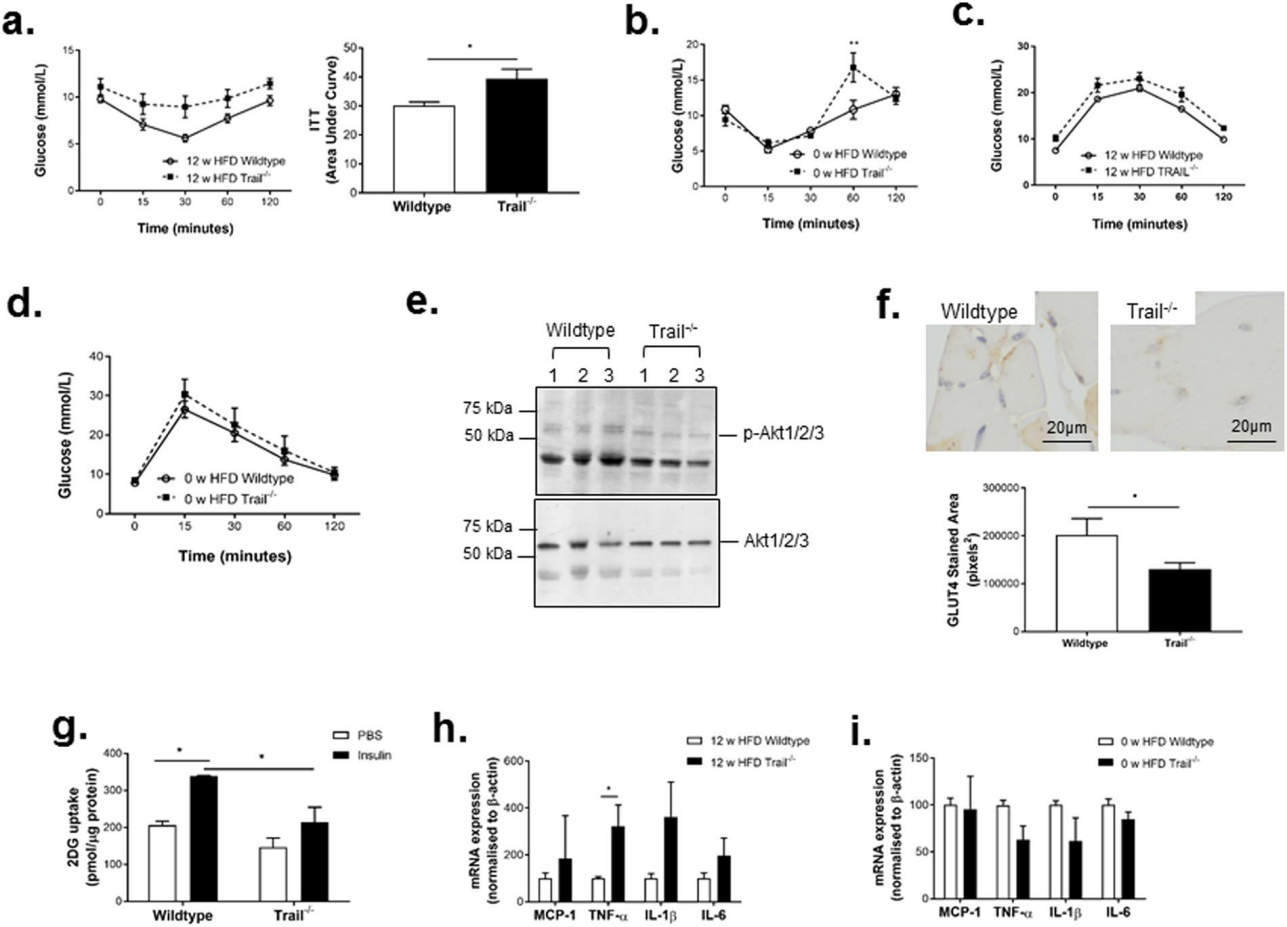
*Trail* ^−*/*−^ mice have impaired insulin sensitivity. (**a**) Insulin tolerance tests (ITT) in 12 w HFD mice (*left panel*); expressed as area under the curve (*right panel*; n = 10/genotype) or, (**b)** ITT at baseline (0 w HFD wildtype and *Trail* ^−*/*−^ mice; n = 3/genotype). (**c**) Glucose tolerance tests in 12 w HFD mice (*left panel*; n = 10/genotype) or, (**d**) at baseline (0 w HFD wildtype and *Trail* ^−*/*−^ mice; n = 3/genotype). (**e**) Western blotting showing that muscle from insulin-stimulated 12 w HFD *Trail* ^−*/*−^ mice have reduced p-Akt protein expression (3 independent mice/group). Total Akt protein expression is unaltered. (**f**) GLUT4 staining in muscle from insulin-stimulated (45 min) 0 w HFD *Trail* ^−*/*−^ and wildtype mice (n = 4/genotype) and (**g**) reduced glucose uptake *ex vivo* (n = 3–4/genotype). (**h**) Inflammatory marker expression is increased in muscle tissue of 12 w HFD *Trail* ^−*/*−^ vs. wildtype, but not at (**i**) baseline (n = 4–5/genotype). mRNA expression was normalized to β-actin. Results are expressed as mean ± SEM; ANOVA or Mann Whitney *U* test. *p < 0.05 and **p < 0.01.

12 w HFD *Trail* ^−*/*−^ mice did not respond to an insulin challenge, such that their plasma glucose levels remained markedly higher over 2 h (Fig. 3a). TRAIL-deletion at baseline (0 w HFD) also resulted in reduced sensitivity to insulin at 60 min following an insulin bolus (Fig. 3b). No change in glucose tolerance was observed (Fig. 3c,d). Skeletal muscle from 12 w HFD *Trail* ^−*/*−^ mice had impaired insulin-induced p-Akt (Fig. 3e), as well as reduced glucose transporter-4 (GLUT4) expression (Fig. 3f) and glucose uptake *ex vivo* at baseline (Fig. 3g). Insulin resistance in skeletal muscle of 12 w HFD *Trail* ^−*/*−^ mice was associated with significantly increased expression of TNF-α (Fig. 3h). No change in monocyte chemo-attractant protein-1 (MCP-1), interleukin-1β (IL-1β), interleukin-6 (IL-6) and TNF-α expression was observed in muscle tissue at baseline (Fig. 3i). Importantly, adipose tissue did not compensate for the skeletal muscle effects, since insulin-inducible glucose uptake was also impaired in WAT *ex vivo* (Supplemental Fig. 2). These findings suggest that the presence of TRAIL improves insulin sensitivity, and that TRAIL-gene deletion impairs insulin signaling and promotes insulin resistance.

### *Trail*^−*/*−^ mice have altered glucose and lipid metabolism

**Figure 4:**
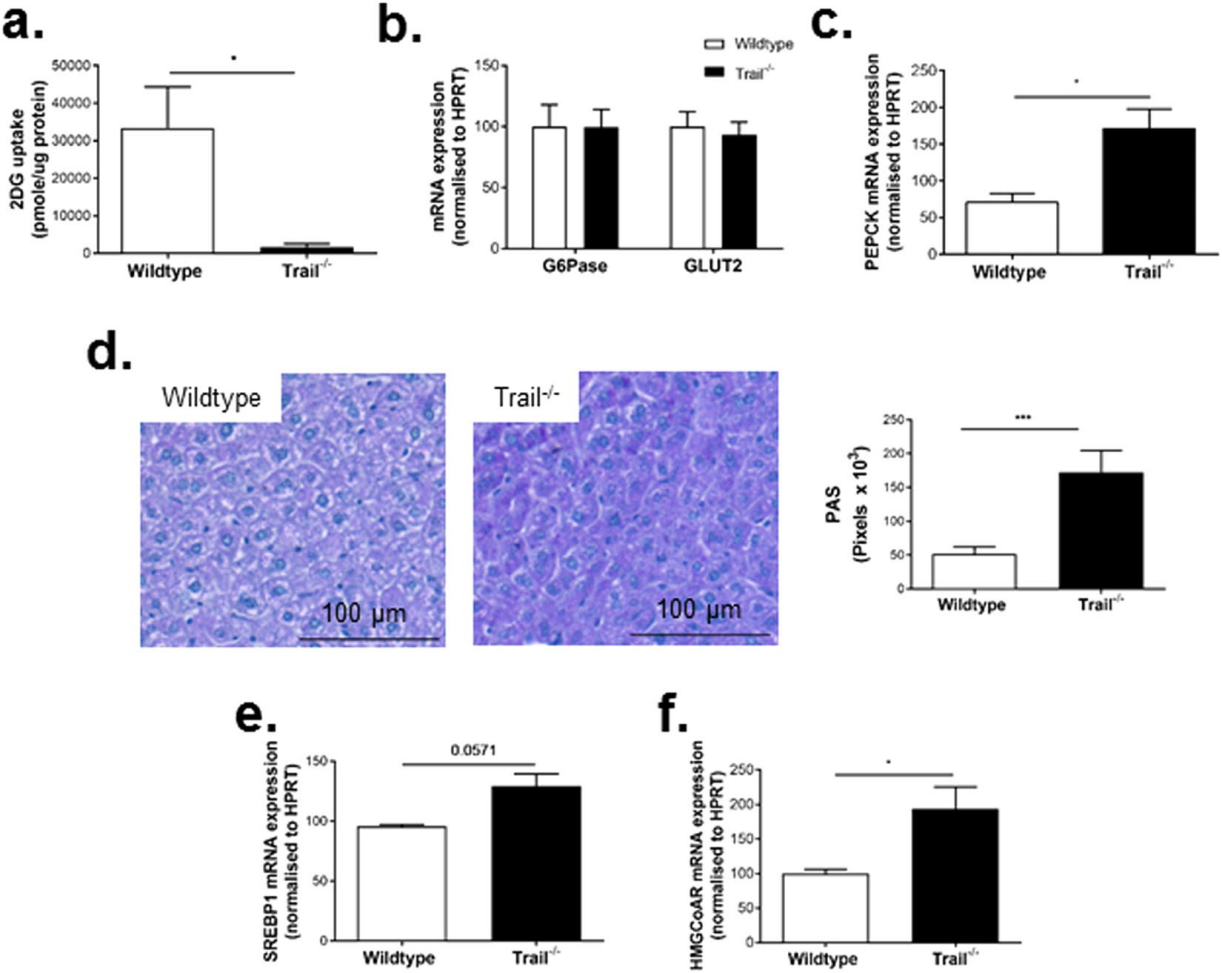
Liver from *Trail* ^−*/*−^ mice exhibit altered glucose and lipid metabolism. (**a**) Hepatic glucose uptake *ex vivo* is reduced in baseline 0 w HFD *Trail* ^−*/*−^ vs. wildtype mice (n = 3–5/genotype). mRNA expression for (**b**) Glucose-6 phosphatase (G6Pase) or GLUT2 mRNA in baseline mice. (**c**) Insulin-stimulated PEPCK mRNA expression is increased in baseline *Trail* ^−*/*−^ mice (n = 6/genotype). (**d**) 12 w HFD *Trail* ^−*/*−^ mice exhibit increased PAS staining and have (**e**) elevated SREBP-1 and (**f**) HMGCoAR mRNA expression (n = 6/genotype). mRNA expression was normalized to HPRT. Results are expressed as mean ± SEM; Mann-Whitney *U*-test; *p < 0.05, and ***p < 0.001.

Given that TRAIL-deletion promoted a T2D phenotype, we next wanted to examine whether hepatic glucose and lipid metabolism was altered in these mice. While glucose uptake in liver was significantly impaired with TRAIL-deletion at baseline (Fig. 4a), no change in glucose-6-phosphatase or GLUT2 expression was observed (Fig. 4b). Baseline *Trail* ^−*/*−^ mice challenged with insulin showed significantly increased hepatic phosphoenolpyruvate carboxykinase (PEPCK) mRNA (Fig. 4c), and increased glycogen content (Fig. 4d). Furthermore, there was a significant increase in hepatic expression of 3-hydroxy-3-methyl-glutaryl-CoA reductase (HMGCoAR; Fig. 4f), with a trend for increased sterol regulatory element-binding protein-1 (SREBP1; Fig. 4e) expression. These data imply that the presence of TRAIL improves hepatic glucose and lipid metabolism.

### Liver from *Trail*^*−/−*^ exhibit altered liver pathology in response to a HFD

**Figure 5:**
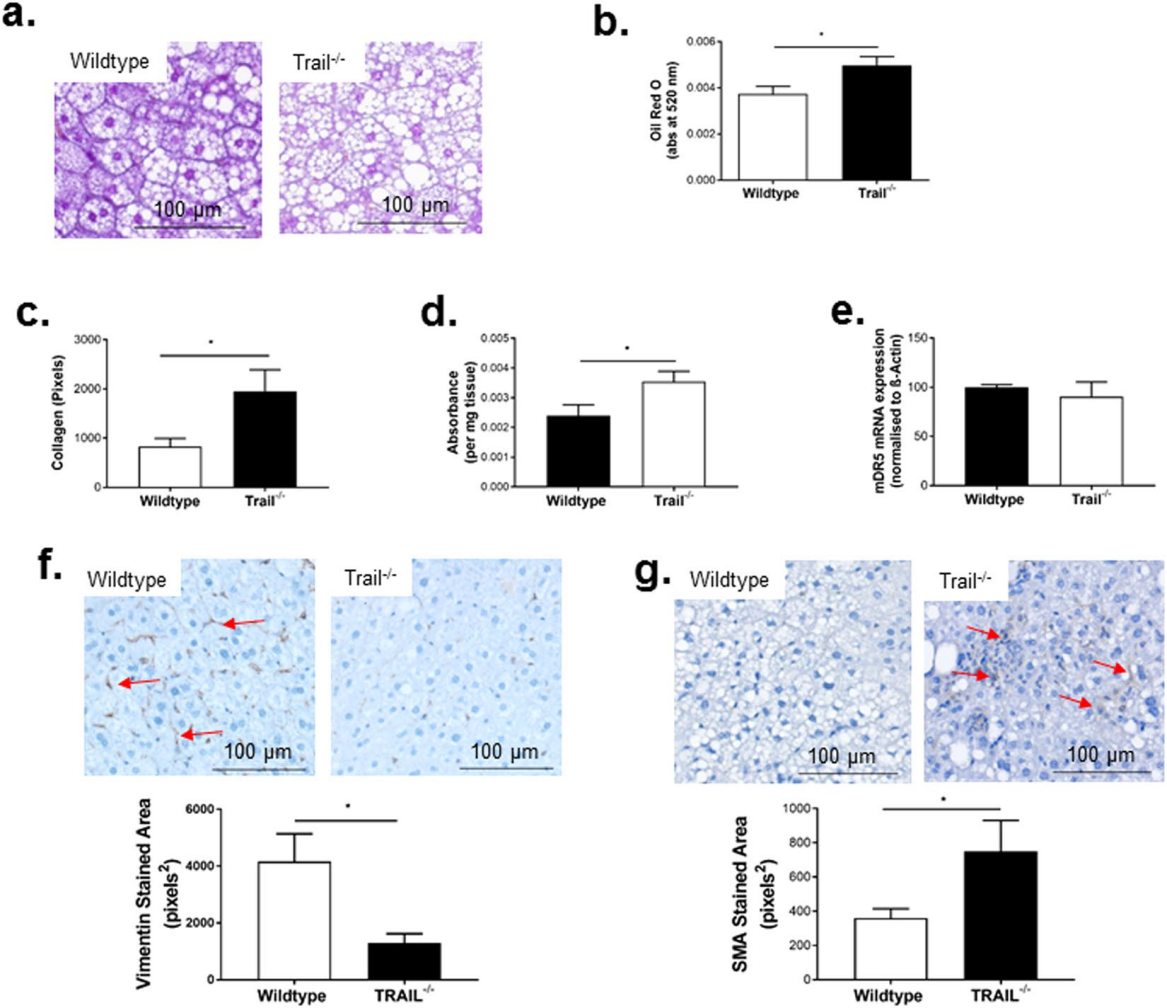
Liver from 12 w HFD *Trail* ^−*/*−^ mice have NAFLD. (**a**) Liver from 12 w HFD *Trail* ^−*/*−^ mice have elevated steatosis compared to 12 w HFD wildtype mice (40× magnification). (**b**) Oil red O is increased in hepatic tissue of 12 w HFD *Trail* ^−*/*−^ vs. wildtype. (**c**) Milligan’s trichrome quantification of fibrosis shows that 12 w HFD *Trail* ^−*/*−^ mice have increased fibrosis. (**d**) Hepatic tissue from 12 w HFD *Trail* ^−*/*−^ mice have increased apoptosis, measured using the Cell Death Detection ELISA. (**e)** mDR5 expression is unaltered in 12 w HFD *Trail* ^−*/*−^ vs. wildtype liver. mRNA expression was normalized to β-actin. (**f**) Vimentin staining is increased in liver from 12 w HFD wildtype mice (*arrows*), while (**g**) SMA staining is increased in 12 w HFD *Trail* ^−*/*−^ liver (*arrows*). Staining was quantified as described in the Materials and Methods. Results are expressed as mean ± SEM (n = 10/genotype); Mann-Whitney *U*-test; *p < 0.05.

We next examined liver of 12 w HFD *Trail* ^−*/*−^ vs. wildtype mice for evidence of NAFLD pathology. Fat engorged hepatocytes were clearly seen in both genotypes (Fig. 5a). Importantly, oil red O staining was significantly elevated with TRAIL deletion (Fig. 5b). 12 w HFD *Trail* ^−*/*−^ liver also displayed increased fibrosis and apoptosis (Fig. 5c,d), with no change in mDR5 expression (Fig. 5e). Hepatic stellate cell activation plays a key role in the development of fibrosis^13^; these cells can be identified by vimentin staining in the quiescent state^14^, or smooth muscle α-actin (SMA) staining in the active state^15^. While vimentin expression was significantly reduced in 12 w HFD *Trail* ^−*/*−^ liver (Fig. 5f), SMA was significantly elevated (Fig. 5g), reflecting a de-differentiation of the cells to a fibroblastic state^15^. Thus, global TRAIL gene deletion in mice exacerbates NAFLD/NASH in response to a HFD.

### Dietary cholesterol is essential for TRAIL-dependent NAFLD/NASH

It has been proposed that increased dietary cholesterol is critical in the development of NAFLD/NASH in humans^16^ and in experimental models^17^. As the HFD employed in this study contains 0.15% cholesterol, we next assessed whether added cholesterol in the diet is critical for TRAIL-dependent NAFLD/NASH. For this we placed wildtype and *Trail* ^−*/*−^ mice on a lard diet for 12 w; containing a similar fat content to the HFD, without cholesterol. While 12 w lard *Trail* ^−*/*−^ mice had significantly increased plasma glucose levels compared to wildtype, no changes in body weight, plasma cholesterol, triglycerides, insulin or NEFA were observed (Supplemental Table 4). No change in GTT or ITT was also evident (Supplemental Fig. 3a,b). Importantly, there was no change in hepatic triglyceride content in these mice (Supplemental Fig. 3c). Collectively, these suggest that dietary cholesterol is essential for the development and severity of NAFLD with TRAIL-deletion.

### *Trail*^*−/−*^ vascular tissue is insulin resistant and displays inflammation

**Figure 6:**
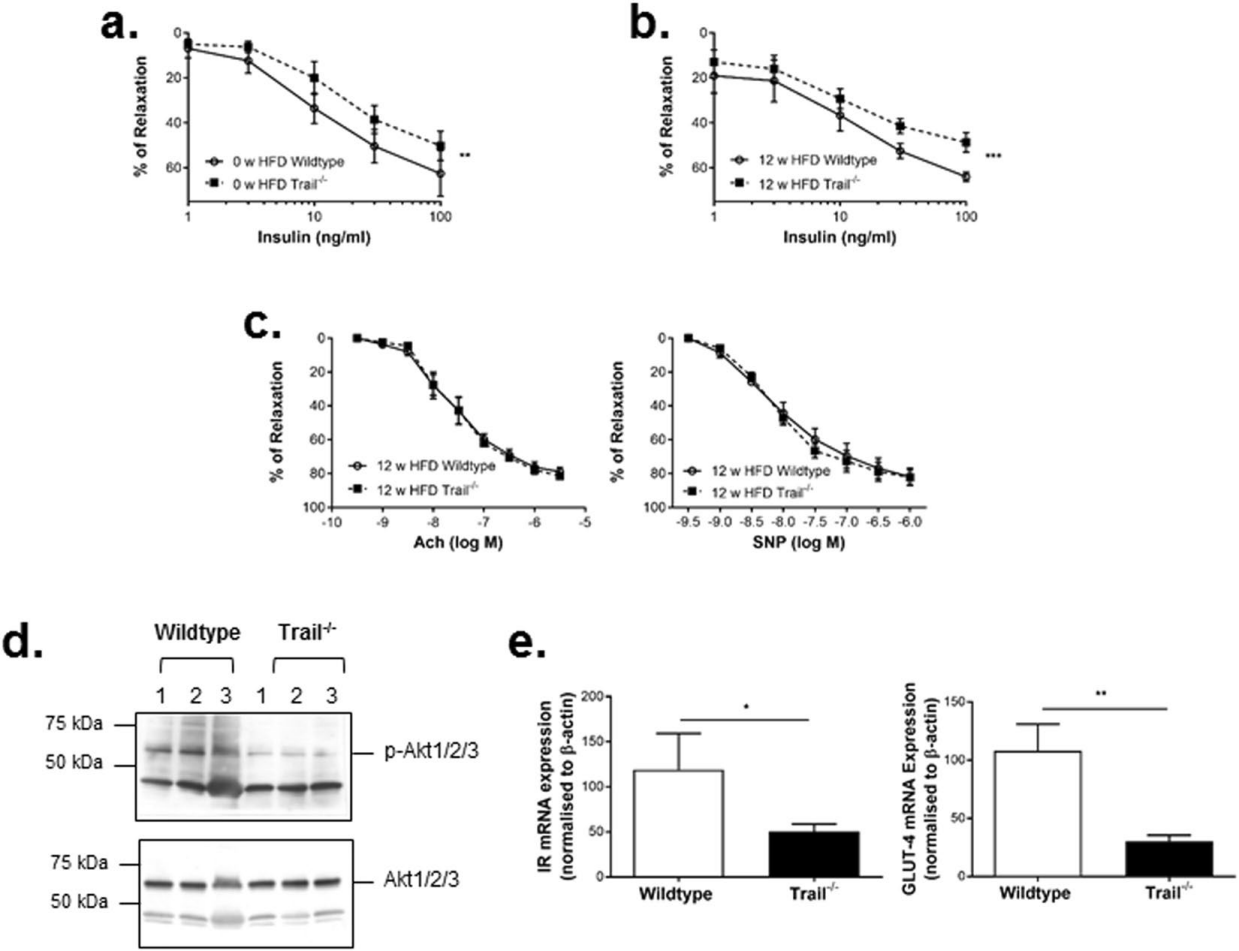
*Trail* ^−*/*−^ vascular tissue have altered insulin signals. Concentration-response curves for *ex vivo* aortic relaxation induced by insulin at (**a**) baseline 0 w HFD or (**b**) 12 w HFD wildtype and *Trail* ^−*/*−^ mice; (n = 5/genotype). (**c**) Concentration-response curves for *ex vivo* aortic relaxation induced by Ach (*left*) and SNP (*right*) in 12 w HFD mice. (**d**) Aortas from 12 w HFD *Trail* ^−*/*−^ mice in response to an insulin challenge (45 min) have reduced p-Akt protein expression, with no change in total Akt levels. (**e**) Aortic mRNA expression of insulin receptor-β and GLUT-4 in 12 w HFD *Trail* ^−*/*−^ vs. wildtype; mRNA levels were normalised to β-actin (n = 3/genotype). Results are expressed as mean ± SEM; ANOVA or Mann-Whitney *U*-test; *p < 0.05, **p < 0.01, ***p < 0.001.

NAFLD is strongly associated with T2D and vascular injury, with CVD an independent risk factor^3^. Insulin-induced aortic vasodilation was impaired with TRAIL deletion at baseline (Fig. 6a), and more profoundly at 12 w HFD (Fig. 6b). In contrast, acetylcholine (Ach) or sodium nitroprusside (SNP)-induced vasodilation was unaltered (Fig. 6c). Importantly, insulin signaling was impaired, with aortic p-Akt expression in response to insulin, markedly reduced in 12 w HFD *Trail* ^−*/*−^ mice (Fig. 6d). 12 w HFD *Trail* ^−*/*−^ mice also had ~50–70% reduced insulin receptor and GLUT4 mRNA (Fig. 6e).

**Figure 7:**
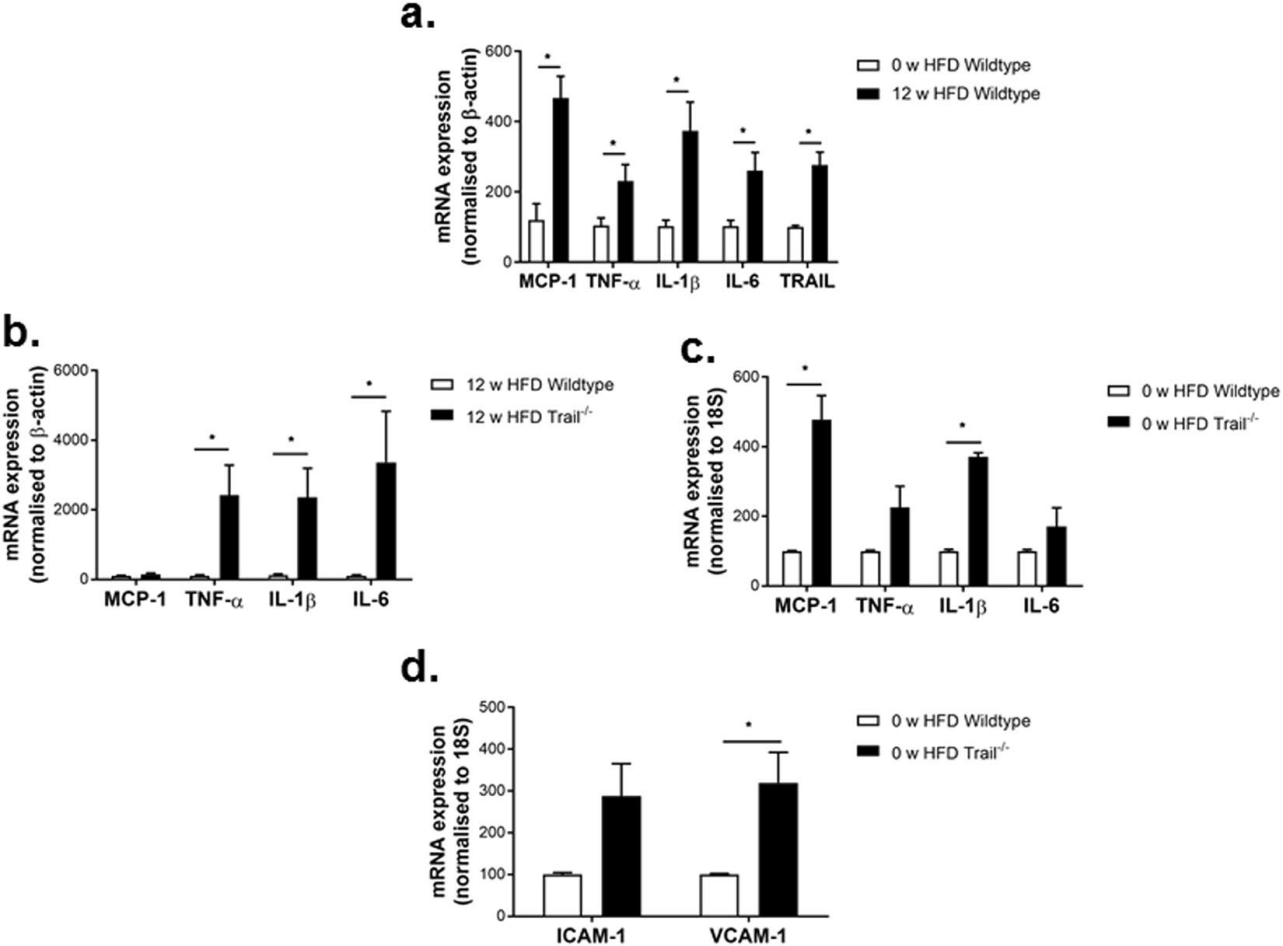
*Trail* ^−*/*−^ mice display severe vascular tissue inflammation in response to a HFD. (**a**) Aortic mRNA expression for MCP-1, TNF-α, IL-6, IL-1β and TRAIL in 0 vs. 12 w HFD wildtype mice (n = 4–5/treatment). (**b**) Aortic mRNA expression for inflammatory markers in 12 w HFD or (**c**) baseline *Trail* ^−*/*−^ vs. wildtype mice. (**d**) Baseline *Trail* ^−*/*−^ have increased aortic mRNA for VCAM-1 but not ICAM-1. mRNA levels were normalized to β-actin or 18S and expressed as mean ± SEM; Mann-Whitney *U*-test (n = 4–5/genotype); *p < 0.05.

The effect of a HFD on TRAIL expression in vascular tissues is not known and low-grade chronic inflammation is important in the development of vascular disease. In response to a HFD, wildtype mice had 2 to 5-fold increases in aortic expression of MCP-1, TNF-α, IL-1β, IL-6 and TRAIL (Fig. 7a). Compared to 12 w HFD wildtype mice however, TRAIL-deletion resulted in marked elevation of aortic TNF-α (22-fold), IL-1β (20-fold) and IL-6 (31-fold) (Fig. 7b). Of note, only vascular MCP-1, IL-1β (Fig. 7c) and VCAM-1 (Fig. 7d) expression were significantly elevated with TRAIL-deletion at baseline. These findings suggest that *Trail* ^−*/*−^ vessels are insulin resistant and have impaired downstream insulin signals. Moreover, *Trail* ^−*/*−^ vessels are more susceptible to inflammation, which is accelerated in response to a HFD.

## Discussion

The key novel findings of this study are: first, that plasma TRAIL levels are significantly reduced in patients with the severe form of NAFLD (i.e. NASH), negatively associating with plasma ALT, even after adjustment for diabetes, BMI, age and sex. Second, in response to a HFD with cholesterol, mice with TRAIL-deletion develop a marked increase in hepatic steatosis, display altered hepatic cholesterol and glucose homeostasis, and many features of NASH. Mechanistically, free-fatty acid overload in hepatocytes was associated with reduced TRAIL mRNA, with TRAIL administration inhibiting lipid accumulation *in vitro*. Third, we show that *Trail* ^−*/*−^ mice are more susceptible to insulin resistance, inflammation, develop T2D and vascular injury in response to a HFD. This is the first report showing that TRAIL protects against hepatic steatosis, inflammation and fibrosis; features of NASH. These findings are significant because increased consumption of energy-dense food has resulted in a pandemic of NAFLD, diabetes and CVD affecting approximately 30% of the world’s population.

The clinical importance of TRAIL in people with NAFLD is uncertain, but does, on balance, favor a beneficial effect. One small study examined soluble TRAIL levels in NAFLD patients from China^18^. This study indicated that the C/G allele polymorphisms in the TRAIL gene at position 1525/1595 were higher in NAFLD than healthy individuals, associating with increased serum TRAIL^18^. On the other hand, the same group showed that the AATT genotype frequencies at 1525/1595 were lower with NAFLD, associating with reduced soluble TRAIL levels^18^. Notably, this report was unable to determine the association between TRAIL levels, NASH and simple steatosis. Here, we show that circulating TRAIL levels are significantly reduced in people with NASH but not with obese individuals, and strongly correlate with levels of ALT.

Hepatic steatosis occurs when there is an imbalance in hepatic fatty acid and triglyceride acquisition and removal. The main sources of free fatty acids in the liver are (i) NEFA released from adipose stores; (ii) *de novo* lipogenesis (e.g. from glucose) involving transcriptional regulation by factors including SREBP-1; and (iii) free fatty acids from dietary intake. Surprisingly, and in contrast to our previous findings in 12 w HFD *Trail* ^−*/*−^ *Apoe* ^−/−^ mice^10^, no changes in food intake, body weight and adipose tissue weight (not shown) were observed between *Trail* ^−*/*−^ and wildtype mice. The surplus lipids observed in *Trail* ^−/−^ *Apoe* ^−/−^ (e.g. plasma cholesterol, triglycerides, LDL and VLDL^10^), may further promote an increase in adipocyte hypertrophy and body weight, not evident with TRAIL-deletion alone. Indeed, excessive lipid can stimulate adipocyte hypertrophy^19^, and promote adiposity in people^20^.

We found no change in plasma NEFA with TRAIL-deletion alone, nor between 12 w HFD *Trail* ^−*/*−^ *Apoe* ^−/−^ vs. *Apoe* ^−/−^ mice (not shown), suggesting that NEFA released from fat stores does not affect hepatic triglyceride levels in these mice. Increases in hepatic HMGCoAR (and to some extent SREBP-1) with TRAIL deletion, imply that cholesterol and triglyceride synthesis is increased in the liver. Moreover, TRAIL-deletion alone impaired the ability of the liver to remove triglyceride, since more was evident by oil red O staining in HFD *Trail* ^−*/*−^ mice. Importantly, these changes in *Trail* ^−*/*−^ mice were only evident in response to a HFD with cholesterol. Of note, dietary fat and cholesterol are known to synergistically interact, enhancing NASH and metabolic changes in mice by an ~2-fold greater extent, than achieved with a fat or cholesterol diet alone^21^; a finding also supported in humans with NASH^16^. Therefore, added dietary cholesterol in a HFD is essential for the NAFLD/NASH we observe with TRAIL-deletion in mice.

In humans, TRAIL signaling occurs upon binding and trimerization of its death receptors, DR4 and DR5. In mice, there is only one TRAIL death receptor, mDR5 with ~60% homology to both DR4 and DR5. Both TRAIL and DR5 are expressed in human hepatocytes *in vitro* ^22^. TRAIL itself can induce apoptosis of normal human hepatocytes at high, non-physiological, concentrations^23^; with free-fatty acids sensitizing human hepatocytes to TRAIL-mediated apoptosis via DR5^24^. Mice fed a fibrogenic methionine and choline-deficient diet develop NAFLD, with an increase in hepatic mDR5 expression and apoptosis^25^, suggesting that mDR5 signals promote a NAFLD phenotype. Furthermore, *Dr5* ^−/−^ mice fed a high fat, high sucrose and high cholesterol diet for 3 months, not dissimilar to our HFD, had reduced hepatic steatosis, inflammation and markers of fibrosis^26^. Fas has also been implicated in NAFLD in people^27^, and in experimental models^28, 29^. Reduced hepatic steatosis was also observed in mice with Fas deletion specifically from adipocytes^30^. These data imply that TNF ligands may promote NAFLD pathogenesis via their cognate receptors. However, the direct effect of TRAIL in experimental models of NAFLD is unknown. We found that global TRAIL-gene deletion in mice promoted a more severe NAFLD phenotype with increased lipid accumulation, inflammation and fibrosis. This apparently contradictory finding may be explained by ligand-independent receptor activation. While no changes in mDR5 mRNA between genotype in the liver was observed, *Trail* ^−*/*−^ hepatic tissues had significantly increased apoptosis. Intriguingly, an overload of lipid in hepatocytes promotes DR5 localization into lipid rafts, stimulating cell death independent of TRAIL binding^31^ and further, ligand independent hepatocyte apoptosis has also been observed for Fas^32, 33^. Whether TRAIL or ligand-independent mechanisms promote liver injury in this setting is unclear and requires further elucidation.

We also found *Trail* ^−*/*−^ mice were insulin resistant, with TRAIL deletion impairing insulin signaling and glucose homeostasis. PEPCK is an enzyme that controls the rate of glucose synthesis with the ability to modulate plasma glucose levels^34^. Overexpression of PEPCK promotes T2D^34^. The increase in plasma glucose levels and glycogen content in *Trail* ^−*/*−^ mice may reflect the role PEPCK plays in regulating hepatic glucose (and cholesterol) homeostasis. Collectively, our study implies that TRAIL improves insulin sensitivity by modulating expression of genes involved in glucose metabolism and insulin signaling. Consistent with this, plasma TRAIL levels in T2D patients after gastric banding surgery were increased and correlated with improved β-cell function^35^. TNF ligand regulation of insulin signaling and glucose homeostasis may in fact be a common theme in diabetics, since TNF-α and FasL have also been implicated in insulin resistance^36, 37^.

Insulin resistance can also develop in cardiovascular tissues where insulin can contribute to the development of CVD, hypertension and metabolic diseases^38^. In wildtype mice, we observed a significant reduction in circulating TRAIL levels in response to a HFD, correlating with an elevation of vascular expression of IL-1β, MCP-1, IL-6 and TNF-α, pro-inflammatory cytokines known to play key roles in insulin resistance and diabetes^39^. In contrast to circulating TRAIL levels, vascular TRAIL expression (including in liver and fat) was significantly increased in these mice. Discrepancies between circulating vs. tissue TRAIL expression have been observed in other inflammatory diseases. For example, plasma TRAIL levels are reduced in patients with chronic kidney disease^40^, and increased TRAIL in kidneys is associated with disease severity in diabetic nephropathy^41^. While this suggests that TRAIL may play an inflammatory role and promote disease, importantly, our global TRAIL knockout studies in nephropathy^42^ and here, demonstrate the opposite. In fact, compared to HFD-fed wildtype mice, TRAIL-deletion resulted in >20-fold elevation of inflammatory marker mRNA in the vessel wall. Increased expression of inflammatory markers was also observed in the liver and in fat (not shown). Thus, *Trail* ^−*/*−^ mice are more susceptible to inflammation, suggesting that early inflammatory events occur with TRAIL deletion alone. Our findings are of major importance. Rather than promoting inflammation and disease, the presence of TRAIL may in fact exert an overall anti-inflammatory action in damaged or injured tissues, resulting in protection against injury.

This study has revealed a critical role of TRAIL in blunting metabolic diseases. Our findings show for the first time that deletion of TRAIL causes systemic insulin resistance, increases hepatic cholesterol and glucose production and renders the liver more susceptible to triglyceride accumulation and injury, promoting a more severe form of NAFLD in response to a HFD. Importantly, these metabolic changes are associated with increased dysfunction and inflammation in blood vessels. Taken together, we speculate that these findings may translate into a therapeutic use for TRAIL as a novel anti-inflammatory agent with vascular protective actions. Indeed, the therapeutic potential of TRAIL in reducing HFD-induced inflammation, adiposity and improving glucose sensitivity in mice has previously been reported^43^. It is therefore possible that in the future, TRAIL and related agents, may offer new therapeutic options for patients with NAFLD, diabetes and CVD.

## Materials and Methods

### Human studies

Adults with biopsy-proven NAFLD, obese individuals with normal liver biopsies or healthy controls were recruited. Informed consent was obtained from all subjects. All methods were carried out in accordance with guidelines and regulations from the National Health and Medical Research Council of Australia; experimental protocols were approved by the Sir Charles Gairdner Hospital Human Research Ethics Committee (2007–098). Healthy controls were defined by the presence of normal liver enzymes and absence of medical conditions requiring medications. Plasma samples were taken following an overnight fast on the day of liver biopsy for patients with NAFLD, or on the day of clinical assessment for healthy controls and frozen at −80 °C. Plasma chemistries were assessed as previously described^44^. Biopsy specimens in NAFLD patients were staged by a liver histopathologist as NASH or non-NASH^45^.

### Animal studies

Male *Trail* ^−*/*−^^10^ and wildtype mice at 6 w of age, weighing 18–20 g were euthanized for baseline studies, and or randomly grouped and placed on a ‘Western’ high fat diet (HFD; SF00–219; 22% Fat, 0.15% Cholesterol Semi-Pure Rodent Diet) or the Lard diet (SF04–001; 23.5% Fat Semi-Pure Rodent Diet) for 12 w; both from Specialty Feeds, Glen Forest, Western Australia. After an overnight fast, mice were anaesthetized by i.p. injection of ketamine (100 mg/kg) and xylazine (10 mg/kg), or isoflurane (2%) via nose cone prior to euthanasia by cardiac exsanguination. Blood was collected and plasma stored at −80 °C. Gastrocnemius muscle, epididymal WAT, liver, and aorta were collected, fixed in formaldehyde for immunohistochemistry (IHC) or snap-frozen for gene and protein expression. All methods involving animals were carried out in accordance with guidelines and regulations from the National Health and Medical Research Council of Australia; experimental protocols were approved under the Animal Care and Ethics Committees at the University of New South Wales (11/71B) or the Sydney Local Health District (2013/049), Sydney Australia.

### Metabolic cages

The Comprehensive Laboratory Animal Monitoring System (Columbus Instruments, Columbus, OH) was used at 12 w HFD. Oxygen consumption (VO_2_) and CO_2_ production (VCO_2_) were normalized to body weight. The respiratory exchange ratio was calculated (VCO_2_/VO_2_). Ambulatory activity was examined where horizontal (XAMB) and vertical (ZTOT) movement were determined. Food intake was measured. All measurements were taken over 24 h.

### Plasma analysis

Plasma glucose was measured using a glucometer (Accu-check Performa, Roche, Mannheim, Germany). Insulin (Mercodia, Uppsala, Sweden), cholesterol, triglycerides, NEFA (all from Wako Diagnostics, Richmond, VA, USA) and TRAIL (human, R&D Systems; murine, USCN Life Science Inc., Houston, USA) were assessed.

### Glucose and insulin tolerance tests

Glucose and insulin tolerance tests (GTT, ITT) were performed as previously described^42^; ITTs were performed in non-fasted mice.

### Myography

Mouse thoracic aortas were isolated, prepared and mounted on the multiwire myography system (Danish Myo Technology, Denmark)^46^. Changes in isometric tension of aortic rings in response to insulin (1–100 ng/ml), acetylcholine (Ach; 0.3–3 μM) or sodium nitroprusside (SNP; 0.3 nM–1 μM) were measured and recorded using PowerLab data acquisition system (AD instruments).

### Ex vivo glucose uptake studies

Soleus muscle, epididymal WAT and liver were isolated from 6 w old mice. Tissues were exposed to recombinant human insulin (100 nM) or vehicle (water) for 15 min, followed by 1 mM 2-deoxyglucose (2DG) for 20 min. 2DG uptake was measured^47^.

### Histology and IHC

Liver sections (3–5 μm) were stained with Milligan’s Trichrome for fibrosis and periodic acid-Schiff (PAS) for glycogen. For assessment of stellate cells, liver were stained for vimentin (1:500, Abcam, Cambridge, UK) and smooth muscle α-actin (SMA; 1:200, Novocastra, Melbourne Australia)^48^. Gastrocnemius muscle isolated from 0 w HFD mice challenged with insulin (1 U/kg, 45 min) were stained for GLUT4 (1:500, Abcam, Cambridge, UK). All control sections with primary antibody omitted were negative. Images were captured using an Olympus BX53 or Zeiss Axio Imager Z2 microscope. % of positive staining/tissue image area was quantified using Image-Pro Premier (Cybernetics, Bethesda, MD, USA)^10^.

### Quantification of lipids from frozen liver tissue

Triglyceride accumulation from frozen liver was quantified as described^49^.

### Tissue Culture

HepG2 cells obtained from ATCC were maintained in Dulbecco’s modified Eagle’s medium supplemented with 10% (v/v) fetal bovine serum, penicillin (5 U/mL), streptomycin (5 μg/mL) (*Lonza*) and 200 mM L-glutamine (*Lonza*) in a humidified atmosphere of 5% CO_2_ at 37 °C. Cells were treated with bovine serum albumin-conjugated palmitate (Seahorse Bioscience, Massachusetts USA) to mimic NAFLD *in vitro* ^11^.

### RNA extraction, cDNA synthesis and qPCR

RNA was extracted from homogenized tissue using the All-Prep DNA/RNA/protein mini kit (Qiagen, Valencia, CA), or TRI reagent (Sigma) for cells. cDNA synthesis and real-time qPCR were performed as described^10, 50, 51^. Relative mRNA expression was normalized to housekeeping genes HPRT, β-actin,18S or GAPDH^52^. Primer sequences are shown in Supplemental Table 5.

### Protein extraction and Western blotting

Protein was extracted from homogenized tissue using the All-Prep DNA/RNA/protein mini kit, or by RIPA buffer. Proteins were resolved on 4–20% gradient gels (Bio-Rad, Sydney, Australia) and transferred onto Immobilon-P PVDF membrane (Millipore, Billerica, MA). Membranes were blocked with 5% skim milk or bovine serum albumin, followed by incubation with primary antibodies; β-actin (15 min, 1:30000, Sigma); insulin receptor-β (overnight, 1:2000, Santa Cruz Biotechnology); Akt1/2/3 (overnight, 1:2000, Cell Signaling) and phosphorylated Akt1/2/3 (p-Akt, overnight, 1:1000, Cell Signaling). Horseradish peroxidase-conjugated secondary antibodies (Dako) were used and signal detected by chemiluminescence (ECL^TM^, Western blotting detection reagent, GE Healthcare, Chalfont St Giles, Buckinghamshire, UK).

### Statistics

Results are expressed as mean ± SEM, unless stated otherwise, and analyzed using GraphPad Prism Version 6.0 (GraphPad Software, San Diego, CA, USA) or SSPS (IBM, version 21.0). Statistical comparisons were assessed with Student *t*-test, Mann Whitney *U*-test, or ANOVA (one- or two-way) with Bonferroni adjustment for multiple comparisons. Chi-squared test was also used where indicated. Bivariate correlation was assessed between human serum TRAIL and clinical and biochemical factors using Pearson or Spearman correlation coefficients, according to the distribution of data, with adjustment of *p* values using Bonferroni correction for multiple testing. The relationship between plasma TRAIL and ALT levels was further evaluated using multivariate linear regression analysis with adjustment for possible confounders. A value of *p* < 0.05 was considered significant.

## Electronic supplementary material

Supplementary information, Supplementary Figs and Tables
