# Supplementary material for: Non-alcoholic fatty liver disease, vascular inflammation and insulin resistance are exacerbated by TRAIL deletion in mice
Source: Sci Rep. 2017 May 15;7:1898. doi: 10.1038/s41598-017-01721-4 (PMC5432513; doi:10.1038/s41598-017-01721-4)
Supplement: Supplementary file 1 — Supplementary information, Supplementary Figs and Tables [file 41598_2017_1721_MOESM1_ESM.pdf]

Non-alcoholic fatty liver disease, vascular inflammation and insulin resistance are exacerbated by TRAIL deletion in mice

Siân P Cartland[1,2], Hanis H Harith[3,4], Scott W Genner[1], Lei Dang[3], Victoria C Cogger[5,6], Melissa Vellozzi[1], Belinda A Di Bartolo[1], Shane R Thomas[3], Leon A Adams[7], Mary M Kavurma[1,2]*.

Supplemental Figure 1

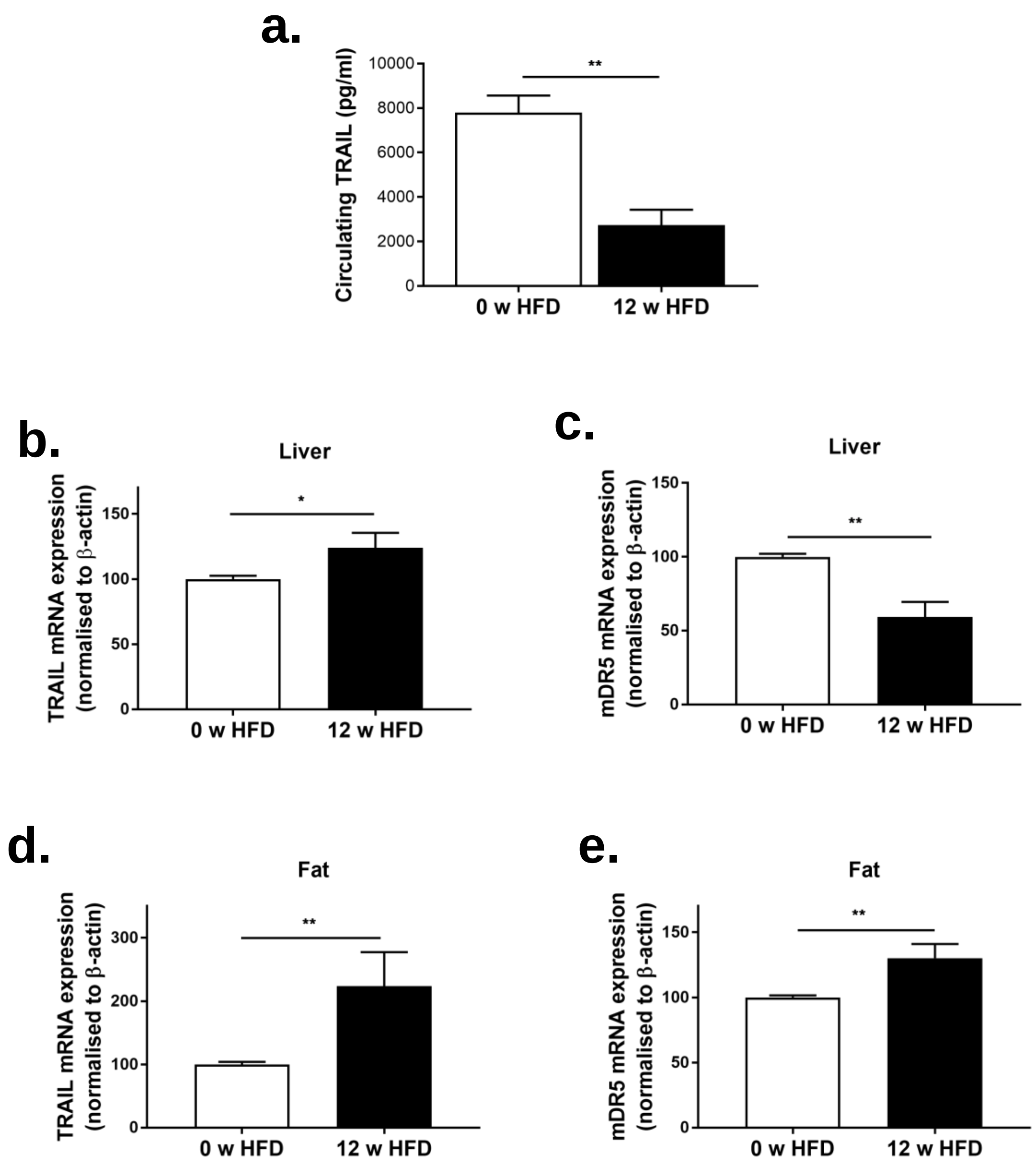

Supplemental Figure 2

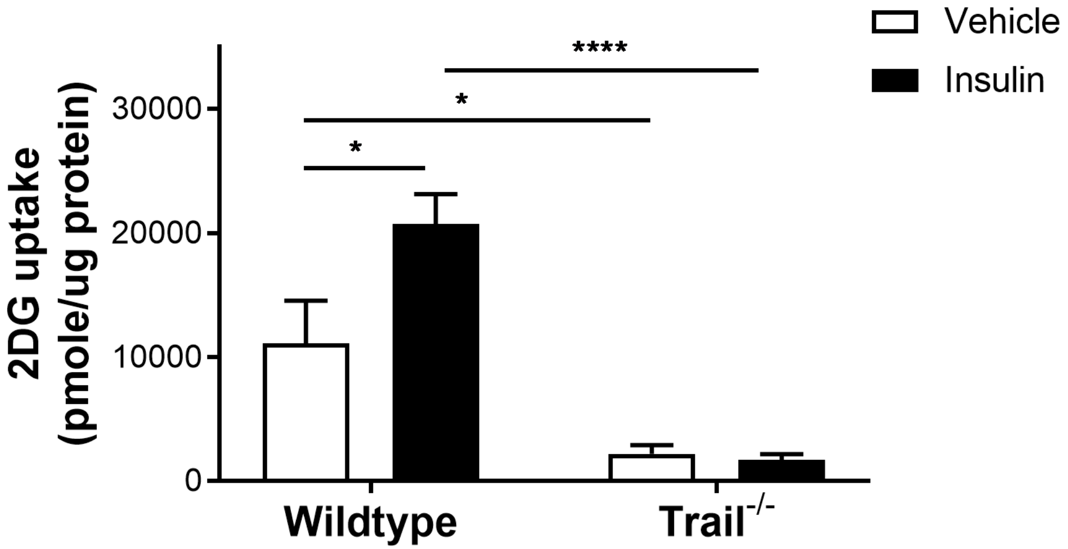

Supplemental Figure 3

a.

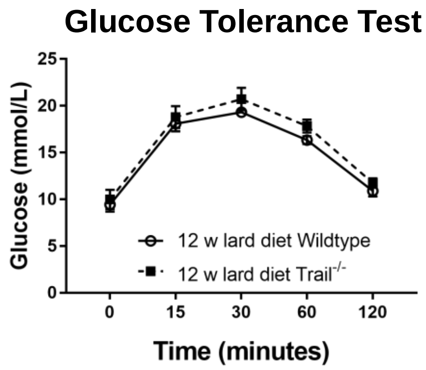

b.

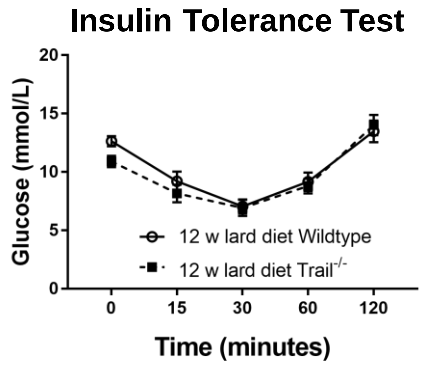

c.

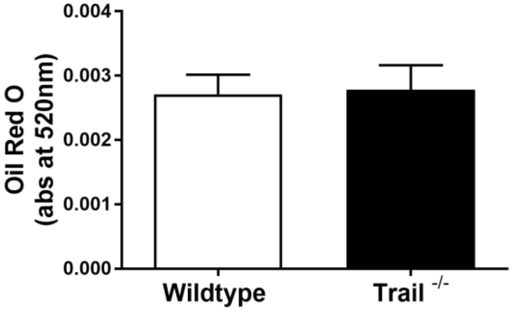

SUPPLENTARY INFORMATION

Supplemental Figure 1. Wildtype mice have reduced circulating TRAIL, but increased TRAIL expression in peripheral tissues. a. Circulating TRAIL levels are reduced in wildtype mice fed a HFD for 12 (n=6/group). Hepatic **b.** TRAIL and **c.** mDR5 expression. WAT **d.** TRAIL and **e.** mDR5 expression. mRNA levels were normalised to β-actin (n=4-7/group). Results are expressed as mean±SEM; Mann Whitney *U* test; **p*<0.05 and ***p*<0.01.

Supplemental Figure 2. WAT from *Trail*$^{-/-}$ mice have impaired glucose uptake *ex vivo*. Insulin-inducible and basal glucose uptake is impaired in baseline *Trail*$^{-/-}$ WAT compared to wildtype (n=5/group). Results are expressed as mean±SEM; ANOVA; **p*<0.05 and *****p*<0.0001.

Supplemental Figure 3. A lard diet has no effect on features of insulin resistance and NAFLD in *Trail*$^{-/-}$ mice. a. Glucose and **b.** insulin tolerance tests in 12 w lard *Trail*$^{-/-}$ and wildtype mice. **c.** Hepatic tryglyceride content as assessed by oil red O is unchanged. Results are expressed as mean±SEM (n=7/genotype).

TABLES

Supplemental Table 1: Correlation between plasma TRAIL and clinical, metabolic and biochemical factors in humans with and without NAFLD.

Factor	Spearman's rho correlation coefficient	*P* value
**Age**	-0.067	0.9
**BMI (kg/m^2)**	-0.174	0.9
**Waist circumference (cm)**	-0.299	0.9
**Glucose (mmol/l)**	-0.184	0.9
**Insulin (U/l)**	-0.116	0.9
**Triglyceride (mmol/l)**	-0.271	0.9
**HDL-cholesterol (mmol/l)**	0.461	0.09
**ALT (IU/l)**	-0.516	0.027
**AST (IU/l)**	-0.458	0.09

Footnote: *p* value with Bonferroni adjustment presented.

Supplemental Table 2: Body weights and plasma chemistries between wildtype and *Trail*$^{-/-}$ mice

	0 w HFD Wildtype	12 w HFD Wildtype	*12 w HFD Trail*$^{-/-}$
**Body weight (g)**	23.5 ± 0.9	33.1 ± 1.3***	33.1 ± 1.0
**Glucose (mmol/L)**	13.4 ± 1.0	17.5 ± 0.9**	23.3 ± 2.0$^{\#}$
**Insulin (pmol/L)**	48.9 ± 3.6	73.2 ± 8.9*	103.3 ± 15.6$^{\#}$
**Cholesterol (mmol/L)**	1.4 ± 0.1	2.0 ± 0.1**	2.5 ± 0.2$^{\#}$
**Triglycerides (mM)**	1.4 ± 0.1	0.9 ± 0.1*	0.6 ± 0.05$^{\#}$
**NEFA (mg/dL)**	0.9 ± 0.1	1.1 ± 0.3	0.6 ± 0.08

Measurements are from n=6-10/genotype; Results are expressed as mean±SEM, Mann-Whitney *U*-test *p<0.05, **0.01, ***0.001 compared to 0 w HFD wildtype; #p<0.05 compared to 12 w HFD wildtype.

Supplemental Table 3: CLAMS data

**12 w HFD**	**Wildtype**	***Trail***$^{-/-}$
**VCO_2 (ml/kg/h)**	92.45 ± 4.758	80.99 ± 3.091
**VO_2 (ml/kg/h)**	109.0 ± 6.578	94.48 ± 3.400
**RER**	0.8491 ± 0.0145	0.8222 ± 0.0121
**XAMB (beam breaks)**	183.4 ± 27.53	278.4 ± 21.200
**ZTOT (beam breaks)**	81.36 ± 5.762	129.0 ± 10.580*
**Food intake (g)**	3.020 ± 0.132	2.782 ± 0.257

Measurements are from n=3-5 mice/genotype. Results are expressed as mean±SEM; Mann-Whitney *U*-test. *p<0.05

Supplemental Table 4: Body weights and plasma chemistries of 12 w lard-fed wildtype and *Trail*$^{-/-}$ mice

	12 w Lard Wildtype	12 w Lard *Trail*$^{-/-}$
**Body weight (g)**	38.81 ± 1.77	37.98 ± 1.51
**Glucose (mmol/L)**	5.95 ± 0.37	9.71 ± 1.18*
**Insulin (pmol/L)**	103.25 ± 21.61	191.23 ± 37.18
**Cholesterol (mmol/L)**	3.06 ± 0.15	2.99 ± 0.10
**Triglycerides (mmol/L)**	0.81 ± 0.08	0.64 ± 0.05
**NEFA (mmol/L)**	0.49 ± 0.06	0.33 ± 0.04

Measurements are from n=7 mice/genotype. Results are expressed as mean±SEM; Mann-Whitney *U*-test. *$p<0.05$ compared to wildtype.

Supplemental Table 5: Primer sequences. m denotes murine; h denotes human sequences.

Gene	Forward primer (5'to 3')	Reverse primer (5'to 3')
mTRAIL	CAGGCTGTGTCTGTGGCTGT	TGAGAAGCAAGCTAGTCCAATTTTG
mDR5	GCAGAGAGGGTATTGACTACACC	GCATCGGGTTTCTACGACTTT
hTRAIL	ACCAACGAGCTGAAGCAGAT	CAAGTGCAAGTTGCTCAGGA
hDR4	CCAACAAGACCTCGCTCCCCAGC	AAGACTACGGCTGCAACTGTGACTCC
hDR5	GTCCTGCTGCAGGTCGTACC	GATGTCACTCCAGGGCGTAC
mInsulin-receptor	TTTGTCATGGATGGAGGCTA	CCTCATCTTGGGGTTGAACT

mIL-1β	GTTTCTGCTTTCACCACTCCA	GAGTCCAATTTACTCCAGGTCAG
mIL-6	CTGCAAGAGACTTCCATCCAG	AGTGGTATAGACAGGTCTGTTGG
mMCP-1	GCTGGAGCATCCACGTGTT	ATCTTGCTGGTGAATGAGTAGCA
mTNF-α	CAGGCGGTGCCTATGTCTC	CGATCACCCCGAAGTTCAGTAG
mVCAM-1	TTGGGAGCCTCAACGGTACT	GCAATCGTTTTGTATTCAGGGGA
mICAM-1	GTGATGCTCAGGTATCCATCCA	CACAGTTCTCAAAGCACAGCG
mG6-phosphatase	CTGTTTGGACAACGCCCGTAT	AGGTGACAGGGAACTGCTTTA
mPEPCK-1	AGCATTCAACGCCAGGTTC	CGAGTCTGTCAGTTCAATACCAA
mSREBP-1	AGCAGCCCCTAGAACAAACAC	CAGCAGTGAGTCTGCCTTGAT
mHMGCoAR	AGCTTGCCCGAATTGTATGTG	TCTGTTGTGAACCATGTGACTTC
mβ-actin	AGCCATGTACGTAGCCATCC	CTCTCAGCTGTGGTGGTGAA
m18S	CGGCTACCACATCCAAGGAA	GCTGGAATTACCGCGGCT
mHPRT	AGTCCCAGCGTCGTGATTAG	TTTCCAAATCCTCGGCATAATGA
hGAPDH	GAAGGCTGGGGCTCATTT	CAGGAGGCATTGCTGATGAT
